# Supplementary material for: Noninvasive vs invasive respiratory support for patients with acute hypoxemic respiratory failure
Source: PLoS One. 2024 Sep 6;19(9):e0307849. doi: 10.1371/journal.pone.0307849 (PMC11379309; doi:10.1371/journal.pone.0307849)
Supplement: S1 Table — (DOCX) [file pone.0307849.s001.docx]

| **S1 Table 1: Demographics** | | | | |
| --- | --- | --- | --- | --- |
| **Measure** | **Invasive Mechanical Ventilation** | **Noninvasive Positive Pressure Ventilation** | **Nasal High Flow** | **Total** |
| N (%) | 1246 (40%) | 1773 (57%) | 77 (2%) | 3096 |
| Female Sex | 565 (45%) | 903 (51%) | 36 (47%) | 1504 (49%) |
| Age, median (IQR) | 61 (47 – 72) | 68 (58 – 77) | 72 (60 – 80) | 66 (54 – 75) |
| BMI, median (IQR) | 28 (23 –- 35) | 29 (23 – 37) | 27 (21 – 34) | 28 (23 – 36) |
| Ethnicity, n(%)^a^ |  |  |  |  |
| Not Hispanic or Latino | 1014 (82%) | 1531 (87%) | 69 (90%) | 2614 (85%) |
| Hispanic or Latino | 222 (18%) | 237 (13%) | 8 (10%) | 467 (15%) |
| Race, n (%)^a^ |  |  |  |  |
| White | 1062 (82%) | 1574 (89%) | 65 (84%) | 2701 (88%) |
| Black or African American | 76 (6%) | 97 (5%) | 4 (5%) | 177 (6%) |
| Asian/Native Hawaiian/Pacific Islander | 16 (1%) | 15 (1%) | 2 (3%) | 33 (1%) |
| American Indian or Alaska Native | 61 (5%) | 34 (2%) | 3 (4%) | 98 (3%) |
| Other | 23 (2%) | 47 (3%) | 3 (4%) | 73 (2%) |
| Hospital Size , n(%) |  |  |  |  |
| small | 39 (4%) | 149 (9%) | 7 (10%) | 195 (7%) |
| medium | 419 (39%) | 761 (47%) | 34 (48%) | 1214 (44%) |
| large | 622 (58%) | 717 (44%) | 30 (42%) | 1369 (49%) |
| APACHE IVa median (IQR) | 68 (50 – 87) | 48 (37 – 62) | 56 (47 – 72) | 56 (43 – 76) |
| Vital Signs on Treatment Assignment median (IQR) |  |  |  |  |
| Heart rate | 93 (79 – 112) | 88 (75 – 103) | 91 (78- 104) | 90 (76 – 107) |
| Systolic blood pressure | 120 (103 – 140) | 132 (116 – 150) | 130 (113 – 143) | 128 (110 – 146) |
| Diastolic blood pressure | 68 (57 – 83) | 73 (64 – 83) | 73 (63 – 85) | 71 (61 – 83) |
| SpO2 | 98 (96 – 100) | 96 (93 – 98) | 94 (92 – 96) | 97 (94 – 99) |
| FiO2^b^ | 70 (50 – 100) | 35 (30 – 50) | 70 (50 – 100) | 45 (33 – 80) |
| SpO2:FiO2 | 131 (99 – 200) | 274 (196 – 327) | 136 (98 – 192) | 209 (121 – 300) |
| Temperature (^o^C) | 37 (36.6 – 37) | 36.8 (36.5 – 37) | 36.7 (36.5 – 36.9) | 36.9 (36.5 – 37) |
| Respiratory Rate | 19 (16 – 23) | 20 (18 – 25) | 20 (18 – 27) | 20 (18 – 24) |
| Comorbidities n(%) |  |  |  |  |
| Diabetes | 460 (40%) | 698 (40%) | 28 (36%) | 1186 (40%) |
| Chronic Kidney Disease | 256 (22%) | 486 (28%) | 19 (25%) | 761 (25%) |
| Heart Failure | 407 (35%) | 839 (48%) | 38 (49%) | 1284 (43%) |
| Hypertension | 825 (72%) | 1330 (75%) | 52 (68%) | 2207 (74%) |
| Chronic Liver Disease | 239 (21%) | 135 (8%) | 9 (12%) | 383 (13%) |
| Neoplasm or Immunosuppression | 170 (15%) | 258 (15%) | 20 (26%) | 448 (15%) |
| COPD | 782 (68%) | 1506 (85%) | 59 (77%) | 2347 (78%) |
| Obesity | 182 (16%) | 338 (19%) | 7 (9%) | 527 (18%) |
| Acute Influenza Diagnosis | 2 (0%) | 3 (0%) | 0 (0%) | 5 (0%) |
| Acute sepsis diagnosis | 413 (36%) | 296 (17%) | 26 (34%) | 735 (25%) |
| Labs on Admission median (IQR) | | | | |
| PaO2 (mmHg) (Worst Value) | 79 (65 – 108) | 76 (63 – 103) | 68 (59 – 89) | 77 (64 – 104) |
| PaO2:FiO2 (Worst Value) | 126 (79 – 221) | 150 (89 – 225) | 76 (61 – 101) | 137 (82 – 221) |
| White Blood Cell Count (K/uL) | 8 (2.5 – 14) | 7.6 (2 – 13) | 8.7 (1.8 – 12) | 7.9 (2.1 – 13.5) |
| Lactate (mmol/L) | 1.9 (1.2 – 3.7) | 1.5 (1 – 2.3) | 1.8 (1.2 – 2.7) | 1.7 (1.1 – 2.8) |
| pH | 7.31 (7.19 – 7.39) | 7.33 (7.26 –- 7.4) | 7.38 (7.34 – 7.45) | 7.32 (7.23 – 7.4) |
| PaCO2 (mmHg) | 46 (38 – 64) | 54 (40 –- 71) | 38 (34 – 49) | 50 (39 – 68) |
| HCO3 (mmol/L) | 24 (20 – 28) | 27 (24 –- 32) | 26 (22 – 29) | 26 (22 – 30) |
| BNP (pg/mL) | 1490 (349 – 5750) | 1276 (323 – - 5315) | 2667 (463 – 7175) | 1350 (328 – 5481) |
| Creatinine (mg/dL) | 1.0 (0.76 – 1.58) | 0.94 (0.73 –- 1.36) | 0.94 (0.75 – 1.3) | 0.97 (0.74 – 1.42) |
| Hospital admission to treatment (h) | 0.59 (-0.67 – 6.58) | 23.4 (0.08 – 84.3) | 78.03 (31.9 – 165.74) | 5.33 (-0.38 – -59.78) |
| Treatment Assignment Location n (%)^a^ | | | | |
| Emergency Department | 660 (53%) | 502 (28%) | 5 (6%) | 1167 (38%) |
| ICU | 493 (40%) | 575 (32%) | 37 (48%) | 1105 (36%) |
| Non-ICU ward | 15 (1%) | 257 (14%) | 10 (13%) | 282 (9%) |
| Stepdown | 78 (6%) | 439 (25%) | 25 (32%) | 542 (18%) |
| ^a^Data are presented as percent of available.  ^b^FiO2 determined by documented FiO2, if documented, or by FiO_2_ = 100(0.21 + oxygen flow [L/min^-1^] x 0.03 if a flow rate was documented. | | | | |
