# Supplementary material for: Noninvasive vs invasive respiratory support for patients with acute hypoxemic respiratory failure
Source: PLoS One. 2024 Sep 6;19(9):e0307849. doi: 10.1371/journal.pone.0307849 (PMC11379309; doi:10.1371/journal.pone.0307849)
Supplement: S2 Table — (DOCX) [file pone.0307849.s002.docx]

**S2 Table: Unmatched outcomes**

| **Outcome** | **Invasive Mechanical Ventilation** | **Noninvasive Respiratory Support** | **Total** | **P-Value** |
| --- | --- | --- | --- | --- |
| All-Cause Hospital Mortality  Total  Failure  Success | 239 (19% of 1266) | 201 (11% of 1911)  105 (27% of 384)  96 (6% of 1527) | 440 (14% of 3177) | p < 0.001  p < 0.001^a^ |
| Failure (intubation rate) |  | 384 (20% of 1911) |  |  |
| Days to Intubation, median (IQR)  From starting noninvasive respiratory support  From hospital admission | --  0.03 (-0.03 - 0.3) | 0.15 (0.07 - 0.35)  0.51 (0.05 - 3.64) | --  0.05 (-0.02 - 0.87) | --  p < 0.001 |
| Duration of Mechanical Ventilation, days, median (IQR) | 1.61 (0.67 - 4.47) | 2.17 (0.88 - 5.49) | 1.77 (0.71 - 4.67) | p = 0.002 |
| Hospital Length-of-Stay  Total  Failure  Success | 6.89 (3.59 - 13.02) | 5.31 (3.1 - 9)  8.21 (4.48 - 15.84)  4.97 (2.96 - 8) | 5.85 (3.25 - 10.14) | p < 0.001  p < 0.001^a^ |
| Estimates are n (% of column n with available data) for categorical characteristics and median (interquartile range) for continuous characteristics. Inferences for categorical variables are the result of Fisher Exact Tests, with p-values computed via Monte Carlo simulation when necessary for computational efficiency. Inferences for continuous variables come from Kruskal-Wallis rank sum test of group differences. ^a^These p-values correspond to tests of outcome distribution differences by failure/success among NIRS patients. | | | | |
