# Supplementary material for: Noninvasive vs invasive respiratory support for patients with acute hypoxemic respiratory failure
Source: PLoS One. 2024 Sep 6;19(9):e0307849. doi: 10.1371/journal.pone.0307849 (PMC11379309; doi:10.1371/journal.pone.0307849)
Supplement: S2 Fig — (DOCX) [file pone.0307849.s004.docx]

**Noninvasive Respiratory Support vs Invasive Mechanical Ventilation Analyses**

**S2 Figure:** Representative In-Hospital Death Model-Estimated Cumulative Incidence Curves Excluding Patients without Clear Sequence of Support


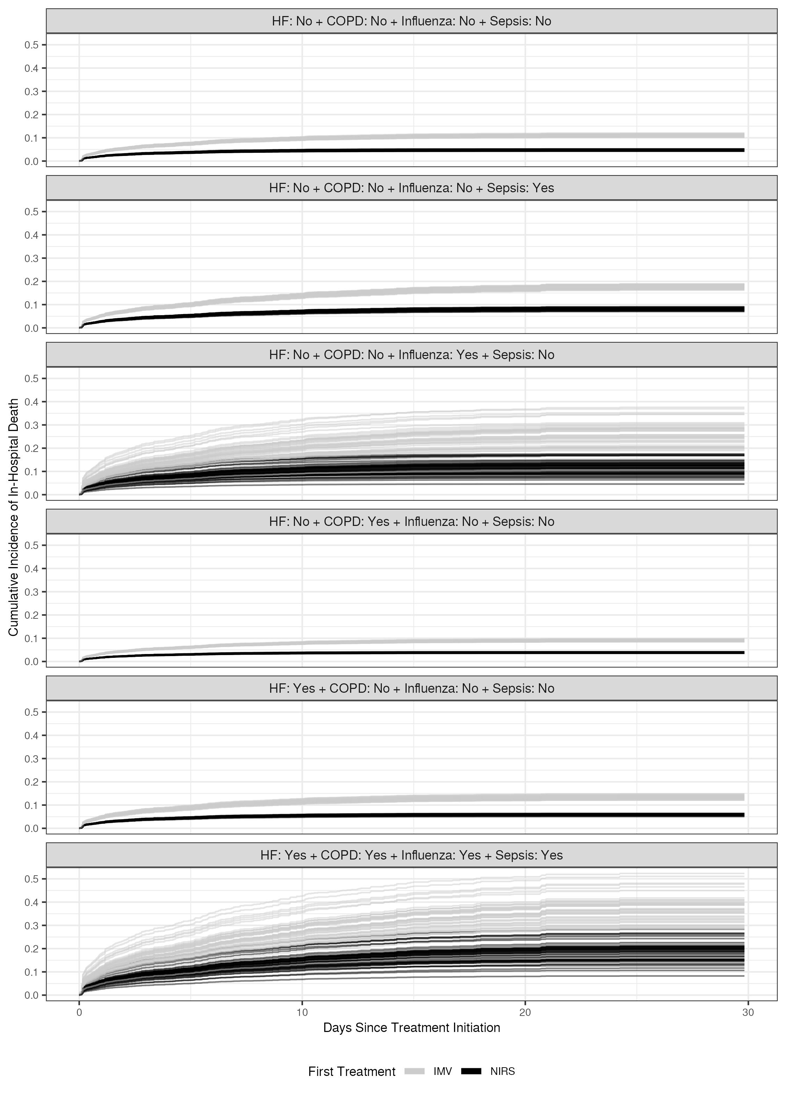


For model-estimated cumulative incidence curves where patients without clear sequence of support have been assigned their temporally first treatment, discrete covariates other than heart failure, chronic obstructive pulmonary disorder, influenza, and sepsis have been set to the following values: male, not Hispanic or Latino, white, one of the large hospitals (hospital A), hospital admission to the emergency department between January 1, 2018 and June 30, 2018, no vasopressor infusion before treatment, no diabetes, no chronic kidney disease, yes hypertension, no neoplasm/immunosuppression, no chronic liver disease, no obesity, and continuous covariates have been set to their median values (age = 66 years, SpO_2_/FiO_2_ = 200, respiratory rate = 20 breaths/min, BMI = 28.44, transformed hours from hospital admission to first treatment = 1.77). For model-estimated cumulative incidence curves where patients without clear sequence of support have been excluded, the only difference in covariate values is that the transformed hours from hospital admission to first treatment = 1.70. For both types of models, the factors heart failure, chronic obstructive pulmonary disorder, influenza, and sepsis have been allowed to vary.
